# The Growth of Yeast and Fungi, the Formation of *β*-Glucan, and the Antibacterial Activities during Soybean Fermentation in Producing Tempeh

**DOI:** 10.1155/2021/6676042

**Published:** 2021-01-25

**Authors:** Samsul Rizal, Maria Erna Kustyawati, Murhadi, Udin Hasanudin

**Affiliations:** Department of Agricultural Product Technology, Faculty of Agriculture, University of Lampung, Jalan Sumantri Brojonegoro No. 1 Bandar Lampung, Lampung 35145 ., Indonesia

## Abstract

Generally, the microorganism involved in soybean fermentation for the production of tempeh is *Rhizopus oligosporus*. However, *Saccharomyces cerevisiae*, a type of *β*-glucan-producing yeast, is known to be present and grow in the fermentation process. This study was aimed at investigating yeast and fungal growth dynamics, *β*-glucan formation, and antibacterial activity against *Escherichia coli* during the fermentation after adding *S*. *cerevisiae* as an inoculum. The Randomized Complete Block Design (RCBD) was applied with two treatments and three repetitions. Three types of starter culture were *S*. *cerevisiae*, *R*. *oligosporus*, and the combination of both. The second treatment was fermentation time at room temperature (30 ± 2°C) for 0, 8, 16, 24, 32, and 40 hours. The dynamics were observed every eight hours. The obtained data were tested using Tukey's Honestly Significant Difference (HSD) test. The results indicated that yeast grew during this process from a single *S*. *cerevisiae* culture and a mixture of *R*. *oligosporus* and *S*. *cerevisiae*, but not from *R*. *oligosporus* alone. The yeast grew during and until the end of fermentation and decreased after 32 hours in the mixed cultures. The *β*-glucan formed in tempeh with all types of inoculum, but the antimicrobial activity against *E*. *coli* increased with fermentation time and was significantly different between treatments. The highest *β*-glucan content and antibacterial activity of tempeh are from the mixed culture. In conclusion, the addition of *S*. *cerevisiae* and *R*. *oligosporus* in soybean fermentation produced tempeh with the highest *β*-glucan content and antibacterial activity against *E*. *coli*. The presence of *β*-glucans suggests higher health benefits of tempeh.

## 1. Introduction

Tempeh is a traditional Indonesian fermented food produced from soybeans by using *Rhizopus* sp. This healthy functional food is due to bioactive compounds such as isoflavones. It has nutritional advantages, unique texture, and pleasant flavors [1]. The quality of tempeh depends on the raw material and type of inoculum or starter culture used. The kind of inoculum plays a vital role in making tempeh because it affects tempeh's quality.

Generally, tempeh uses an inoculum containing *R*. *oligosporus* [2]. Other important microorganisms involved in fermenting soybeans to form tempeh are *R*. *oryzae* and *R*. *stolonifer* [3]. All three microorganisms ferment soybeans into tempeh. *Rhizopus oligosporus* retains most of the nutrients in soybeans and increases protein digestibility [4]. *R*. *oligosporus* synthesizes more protease enzyme, whereas *R*. *oryzae* favors the *α*-amylase enzyme [5].

Besides *R*. *oligosporus*, yeast and bacteria were also involved during fermentation and significantly contributed to producing functional metabolites [6]. Seumahu et al. [7] and Efriwati et al. [8] found lactic acid bacteria (LAB) and yeast in tempeh. A kind of yeast found in tempeh fermentation was *Saccharomyces cerevisiae* [9], which is known as a *β*-glucan-producing microorganism [10].

In this study, *S*. *cerevisiae* was added intentionally to the soybean fermentation process to produce tempeh with high *β*-glucan content and, therefore, to improve the functional properties as a healthy food. The *S*. *cerevisiae* cell wall is composed of *β*-(1,3)- and *β*-(1,6)-glucan, mannan, chitin (1-2%), and mannoproteins, comprising about 20-30% of the dry weight of the cell wall [11]. *β*-Glucan is a polysaccharide that has health benefits, for example, as a biological response modifier [12] and as an antibiotic against bacteria, fungi, viruses, and parasites [13].

The yeast could grow alongside fungi during soybean fermentation when a carbon source was added, thus resulting in *β*-glucans in the tempeh produced [14]. In this study, *S*. *cerevisiae* was added to the soybean fermentation without carbon sources. It is important to test whether the addition of fungi without any carbon source in the fermentation of tempeh can provide yeast growth and the formation of *β*-glucans in tempeh. Also, the presence of *β*-glucan due to yeast addition might add health benefits, including antibacterial activity. Therefore, this study was aimed at observing the effect of *S*. *cerevisiae* addition to the growth dynamics of yeast and fungi, the *β*-glucan formation, and the antibacterial activities against *Escherichia coli* during soybean fermentation to produce tempeh.

## 2. Materials and Methods

This study used pure cultures of *R*. *oligosporus* FNCC 6010, *S*. *cerevisiae* FNCC 3012, and *E*. *coli* obtained from the UGM Inter-University Food and Nutrition Center, soybeans (brand “Soybean USA No. 1”), Nutrient Broth (NB), Nutrient Agar (NA), Malt Extract Agar (MEA), and Potato Dextrose Agar (PDA). The experimental analysis employed a Factorial Randomized Complete Block Design with three repetitions. The first factor was the three inoculum treatments: *S*. *cerevisiae*, *R*. *oligosporus*, and the mix of both microorganisms. The second factor was the fermentation time of six levels: 0, 8, 16, 24, 32, and 40 hours. During fermentation time, we observed the microbial population, the *β*-glucan content, and the antibacterial activity toward *E*. *coli* in (0, 8, 16, 24, 32, and 40 hours) of fermentation time. The obtained data were tested using Tukey's Honestly Significant Difference (HSD) test.

### 2.1. Preparation of *S*. *cerevisiae* Culture

The *S*. *cerevisiae* was cultured in a sterile Malt Extract Agar (MEA) medium using a sterilized inoculating loop needle with a scratchplate, then incubated for 24 to 48 hours at 28°C to form colonies. The colonies were harvested by adding 5 or 10 mL of distilled water into the plate disc. The fungal cells were harvested and poured into a 50 mL centrifuge tube. The tube was weighed and spun at 3000 rpm for 10 minutes to obtain a separate solid from the supernatant. The supernatant was discarded, and the remaining solids were diluted with 25 to 30 mL of distilled water. The cells were transferred into a test tube containing 9 mL of physiological saline solution and then homogenized using a vortex. The number of cells was calculated using a hemocytometer. The required concentration was 10^7^ cells/mL.

### 2.2. Preparation of *R*. *oligosporus* Culture

*R*. *oligosporus* from a tilted agar was cultured in a sterile medium of Potato Dextrose Agar (PDA) using a sterilized inoculating loop needle and a scratchplate. The mold was incubated for five to seven days at 30 to 35°C to obtain pure colonies, harvested in the same way as the *S*. *cerevisiae*. The required concentration was 10^5^ cells/mL, 100 times less than *S*. *cerevisiae*.

### 2.3. Production of Soybean Tempeh

300 g of soybeans was immersed in water at room temperature (30 ± 2°C) overnight, then boiled in water with a ratio of 1 : 3 soybean to water for 30 minutes, drained, cooled to ambient temperature, and inoculated with starters.

Three separate 100 g samples of boiled soybeans received these inoculums:

1. 1 mL suspension of 10^5^ spores/mL of *R*. *oligosporus*
2. 1 mL suspension of 10^7^ cells/mL of *S*. *cerevisiae*
3. 1 mL suspension of 10^5^ spores/mL of *R*. *oligosporus*+1 mL suspension of 10^7^ cells/mL of *S*. *cerevisiae*

The samples were packaged in plastics perforated for ventilation, then incubated at 32°C for 40 hours, and observed every eight hours.

### 2.4. Enumeration of Microorganisms

The microorganisms were enumerated by culturing on PDA for the fungi and MEA for the yeast. Immediately at 0 hours, then at 8, 16, 24, 32, and 40 hours, consecutively, each tempeh was sampled and diluted following the method of Kustyawati [20]. Ten grams of the sample and 90 mL of peptone water were homogenized with a stomacher paddle blender for five minutes, then diluted into the concentration series. One mL of each dilution was planted with the appropriate surface plate calculation method on the media. Incubation continued for 24 to 48 hours at 32°C to grow fungi and 30°C to grow yeast.

### 2.5. Analysis of *β*-Glucan

The *β*-glucan formation was analyzed every eight hours during fermentation following Rizal et al. [14]. One gram of the sample and 30 mL of 0.7 N NaOH were hydrolyzed for six hours at 75°C and centrifuged at 10,000 rpm at 25°C for 30 minutes. The supernatant was removed, and the residue was washed with 30 mL of 0.5 M acetic acid solution and centrifuged again at 10,000 rpm and 25°C for 30 minutes. This process was repeated three times. The precipitated material was washed twice with 20 mL of water and centrifuged at 5,000 rpm for 10 minutes.

The residue with 20 mL of ethanol was centrifuged at 5,000 rpm for 10 minutes, resulting in wet *β*-glucan (crude). This biomass was dehydrated at a 45°C oven for 24 hours and weighed to obtain the dry weight of *β*-glucan (crude). The dry residue with 4 mL of 1 M NaOH was left for one hour. Afterward, the sample was diluted with 10 mL of sterile distilled water and shaken with an orbital shaker. The sample was added with 2 mL of Pb acetate and left to stand for 30 minutes. Finally, one gram of sodium oxalate clears the solution, and two mL of it with 0.5 mL of phenol 5% and 2.5 mL of sulfuric acid 5 N was tested using a sugar-free content spectrophotometer under 490 Å wavelength.

### 2.6. Assessment of Antibacterial Activities

#### 2.6.1. Preparation of *E. coli*

Pure *E*. *coli* (20 *μ*L) were grown on Mac Conkey Agar (MCA) media and incubated at 37°C for 24 hours. The bacteria were taken with an inoculating loop needle from the MCA media and put into the Nutrient Broth (NB) media and incubated at 37°C for 24 hours. One mL of the bacterium was diluted in 9 mL of physiological NaCl 0.85% in a sterile test tube and homogenized using a vortex for 15 seconds.

#### 2.6.2. Antibacterial Testing

100 *μ*L of the bacteria was poured evenly on the surface of the NA medium using the spread plate method and let dry. A 2 g sample from each treatment was dissolved in 8 mL of sterile distilled water. A paper disc (5.5 mm diameter) was inserted into each of these treatments and allowed to stand for 10 minutes. After that, the paper disc was placed on the NA medium's surface containing the target bacteria, then incubated at 37°C for 24 hours. After 24 hours, the inhibitory area diameter formed surrounding the paper disc was measured using a slide. The sample's antibacterial activity was expressed by the inhibitory zone diameter as a clear area around the disc. The antibacterial testing was carried out in 3 repetitions.

## 3. Results and Discussion

### 3.1. Growth of Yeast and Fungi during Fermentation

**Figure 1:**
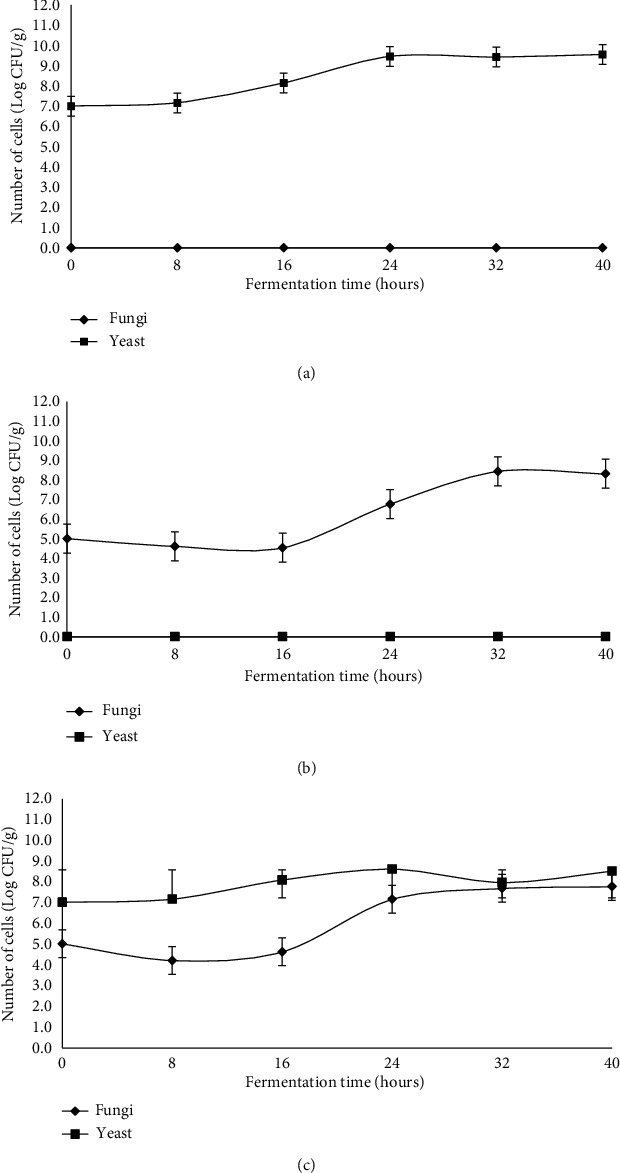
The growth curves of yeast and fungi during soybean fermentation inoculated with a single culture of *S*. *cerevisiae* (a), a single culture of *R*. *oligosporus* (b), and a mixed culture of both microorganisms (c).

Figure 1 shows the growth of yeast and fungi in various types of cultures used in the fermentation of soybeans to tempeh. During fermentation of soybeans with only the *R*. *oligosporus* culture, there was no increase in the amount of yeast (Figure 1(a)), whereas during fermentation using only *S*. *cerevisiae* alone, the fungus did not grow (Figure 1(b)). In contrast, both fungi and yeast reproduce well during the fermentation of soybeans with mixed cultures of *R*. *oligosporus* and *S*. *cerevisiae* (Figure 1(c)).

Figure 1(a) shows the growth curve of *S*. *cerevisiae* in tempeh inoculated with only *S*. *cerevisiae*. The adaptation phase occurred in zero up to 8 hours of fermentation with a population of 10^7^ CFU/g. For comparison, Sugoro and Pikoli [15] stated that the adaptation phase in a modified 1% tapioca solution medium containing 10.21% glucose was at the sixth hour of fermentation. Kusmiati et al. [16] reported that in media with glucose as a carbon source, this fungus' adaptation phase was four hours. On the YNB medium containing 30% of glucose, Ishmayana et al. [17] had it at six hours of fermentation. Our adaptation phase in this experiment was delayed than those of Sugoro and Pikoli [15], Kusmiati et al. [16], and Ishmayana et al. [17] because there was no carbon source on the substrate as needed for the growth during fermentation.

Figure 1(a) also shows that after eight hours of fermentation, the yeast experiences a sharp increase in the number of cells from 1.73 × 10^8^ CFU/g at 16 hours of fermentation to 3.33 × 10^9^ CFU/g at 24 hours of fermentation. This increase indicated that yeast (*S*. *cerevisiae*) entered an exponential phase after eight hours. Kavanagh [18] stated that in the exponential phase, the yeast reproduced by budding. The maximum specific growth rate (*μ*_max_) of yeast is 0.012 cells/hour based on the exponential phase. Furthermore, the yeast experienced a stationary phase from 24 hours to 40 hours, with a population of 4.82 × 10^9^ CFU/g. The death phase of yeast appeared to occur after 40 hours of fermentation time.

**Figure 2:**
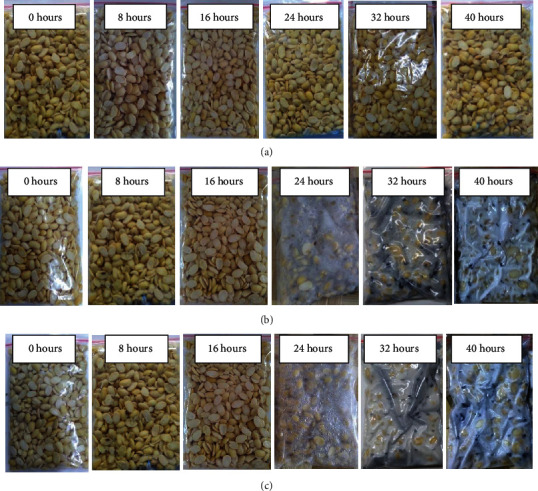
The appearance of soybeans inoculated with a single culture of *S*. *cerevisiae* (a), a single culture of *R*. *oligosporus* (b), and mixed culture of both microorganisms (c) during tempeh fermentation.

Yeast can grow during the fermentation process of soybeans inoculated with only *S*. *cerevisiae* even though tempeh is not formed. *S*. *cerevisiae* as a sole culture (without the addition of the primary tempeh fungus) in 40 hours of fermentation does not form tempeh (Figure 2(a)). To make tempeh, an inoculum is needed. Otherwise, the soybeans will simply decay. *S*. *cerevisiae* increases, but there is no presence of *R*. *oligosporus* unless it is inoculated. This result is in line with Wahono et al. [19], who reported that during the fermentation of sorghum seeds in bioethanol production, there was an increase in the growth rate of *S*. *cerevisiae*. Yeast can grow by utilizing the nutrients present in the soybean substrate. According to Kustyawati (2010), almost all foods provide sufficient nutrition to support yeast growth.

Figure 1(b) shows no growth of yeast during tempeh fermentation with *R*. *oligosporus* as a single inoculum. Unless *S*. *cerevisiae* is inoculated, there will be no yeast growth. In soybean fermentation using *R*. *oligosporus* as a single culture, there was no yeast growth, but tempeh was still formed due to hyphae from *R*. *oligosporus* (Figure 2(b)). This result was in line with Kustyawati [20], which stated that yeast was not found during tempeh fermentation using *R*. *oligosporus*. Thus, this study revealed that yeast in tempeh could only be seen when the fermented soybeans were added with yeast.

The growth dynamics of yeast and the appearance of soybeans during tempeh fermentation inoculated with the mixed culture of *R*. *oligosporus* and *S*. *cerevisiae* are presented in Figures 1(c) and 2(c). Figure 1(c) shows that yeast's adaptation phase occurs between zero and eight hours while the adaptation phase of fungi occurs between zero and 16 hours. In this sample, both microorganisms grew simultaneously and continued increasing until the end of the experiment at 40 hours of fermentation. The appearance of tempeh inoculated with mixed cultures of *R*. *oligosporus* and *S*. *cerevisiae* during fermentation showed that there was no significant fungal growth from 0 to 16 hours of fermentation and soybeans were still intact (Figure 2(c)). After 16 hours of fermentation, fungi entered the exponential growth phase marked by an increase in the number of *R*. *oligosporus* spores up to 7.67 × 10^6^ CFU/g at 24 hours of fermentation time and 2.73 × 10^7^ CFU/g at 32 hours of fermentation time.

This growth pattern is in line with the growth pattern of *S*. *boulardii*, which was inoculated together with *R*. *oligosporus* for tempeh fermentation in a study conducted by Kustyawati [20]. The yeast growth pattern in this treatment was similar to that of soybeans inoculated with *S*. *cerevisiae* alone. It indicates that *S*. *cerevisiae* utilizes the nutrients present in soybeans for growth, and there is a mutually beneficial symbiosis between *R*. *oligosporus* and *S*. *cerevisiae* during fermentation. According to Kustyawati [20], there may be a mutually helpful symbiosis in nutrient availability between *R*. *oligosporus* and *S*. *cerevisiae* during tempeh fermentation to achieve synergistic growth. *Rhizopus oligosporus* breaks down carbohydrate, fat, and protein into simple forms, and *S*. *cerevisiae* absorbs the elements C, H, O, and N from them. In turn, enzymatic activity by *S*. *cerevisiae* benefits *R*. *oligosporus*.

### 3.2. Formation of *β*-Glucan

**Figure 3:**
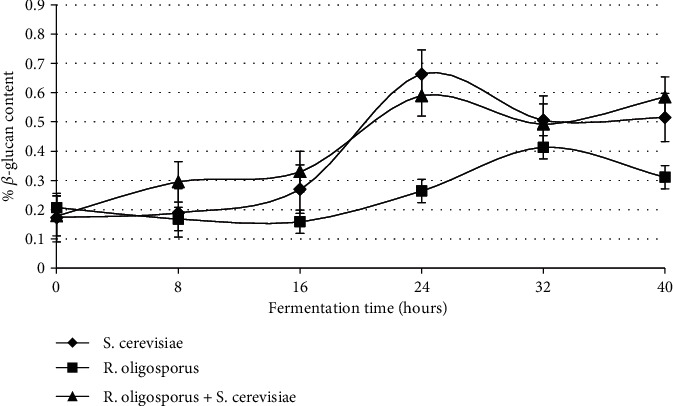
The *β*-glucan content of soybeans fermented using three kinds of starter culture.

*β*-Glucans are polysaccharides that have several health benefits, including as an antimicrobial [13], which can inhibit the growth of bacteria and viruses. All types of starter culture increased the *β*-glucan content of tempeh over time (Figure 3). The *β*-glucan content of tempeh was higher (0.05% *w*/*w*), compared to that without inoculum. Soybeans inoculated by *S. cerevisiae* and mixed inoculums produced higher *β*-glucans in tempeh compared to soybeans without inoculums.

*β*-Glucan can be taken from the *S*. *cerevisiae* cell wall by alkaline extraction, but further purification is needed [21]. The commercial tempeh inoculum contains not only *R*. *oligosporus* but also other microorganisms and fillers such as rice flour [22]. The *β*-glucan content depends on the addition of *S*. *cerevisiae* [23] because its cell wall contains *β*-(1,3)- and *β*-(1,6)-glucans [11].

Soybeans inoculated with *S*. *cerevisiae* contained more *β*-glucan than those without *S*. *cerevisiae* (Figure 3). These results agreed with Thontowi et al. [24] that the *β*-glucan content of *S*. *cerevisiae* in cultures with N peptone sources tended to increase along with fermentation time and was relatively constant by the end of fermentation time (84 hours). Kusmiati et al. [16] also reported an increase in *β*-glucan production using different carbon sources, for example, by utilizing sugar mill waste (molasses) as a fermentation medium. Increased *β*-glucan production follows the increasing number of *S*. *cerevisiae* cells. The formation of *β*-glucans continues until *S*. *cerevisiae* reaches a stationary growth phase. Kim et al. [25] reported that the *β*-glucan content of polysaccharides in black rice bran fermented by *L*. *edodes* increases with time.

Figure 3 shows that the *β*-glucan content of tempeh (0.578%) from this study is higher than that from Rizal et al. [26] with 0.076%. Shokri et al. [27] obtained *β*-glucan from *S*. *cerevisiae* cell walls using NaOH with 27.5% of *β*-glucan, whereas Varelas et al. [28] got 40% of *β*-glucan. Meanwhile, our percentages ranged from 0.05% to 0.663%, significantly lower than the numbers previously mentioned. This difference was caused by the different methods used to extract *β*-glucan. In this study, the *β*-glucan content was investigated from the resulting fermented soybean flour, while the *β*-glucan content was observed by Shokri et al. [27] and Varelas et al. [28] and was directly isolated from the cell wall of *S*. *cerevisiae*.

This study showed that the addition of *S*. *cerevisiae* in the making of tempeh could increase yeast growth and *β*-glucan content of tempeh. The highest content of *β*-glucan was found in tempeh, which was made by adding a mixed culture of *R*. *oligosporus* and *S*. *cerevisiae* inoculums at a fermentation time of 40 hours (0.578% *w*/*w*) (Figure 3).

### 3.3. Antimicrobial Activities of Tempeh during Fermentation

Antibacterial activity testing was carried out during the fermentation process of soybeans added with various cultures (soybeans+*S*. *cerevisiae*, soybeans+*R*. *oligosporus*, and soybeans+*S*. *cerevisiae*+*R*. *oligosporus*). In this study, tempeh's antibacterial activity was determined by measuring the inhibitory zone diameter in the form of a clear area around the paper disc. The results showed that tempeh's antibacterial activity increased along with fermentation time for all treatments of starter culture types. The highest inhibitory zone appeared in tempeh fermented by the mixed starter culture at 40 hours of fermentation time, 25.98 ± 0.56 mm. Meanwhile, the lowest inhibitory area diameter was in soybeans without added inoculum with 7.68 ± 0.39 mm. The diameters of the tempeh inhibitory area against *E*. *coli* are different in various cultures and fermentation times (Figure 3).

**Figure 4:**
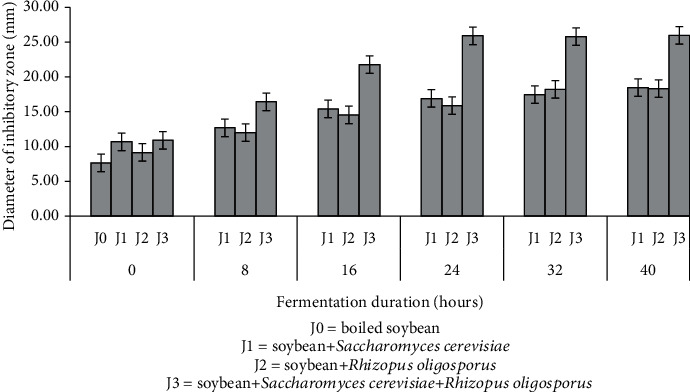
Antibacterial activities of soybeans inoculated by a culture of *Saccharomyces cerevisiae*, a culture of *Rhizopus oligosporus*, and a mixed culture of both during tempeh fermentation.

Figure 4 shows that the boiled soybeans without any starter culture addition could still inhibit the growth of *E*. *coli* with an inhibitory area diameter of 7.68 ± 0.39 mm. The content of isoflavones in soybeans causes the antibacterial activity of soy. According to Kustyawati [20], antibacterial activity happens because soybeans alone contained isoflavones in the form of genistein (0.25 ± 0.60) and daidzein (0.69 ± 0.20). Additionally, according to Dhayakaran et al. [29], soy isoflavones also show antibacterial activity against several pathogens such as *Listeria monocytogenes* and *Pseudomonas aeruginosa*.

The addition of soybeans with all three types of starter culture caused an improvement in antibacterial activity during fermentation that continued to increase along with fermentation time. Both *S*. *cerevisiae* and *R*. *oligosporus* contribute to increase antibacterial activity during tempeh fermentation. The highest antibacterial activity was in tempeh added with mixed cultures of *S*. *cerevisiae* and *R*. *oligosporus* after 40 hours of fermentation. The increase in tempeh antibacterial activity during soybean fermentation by *S*. *cerevisiae* and *R*. *oligosporus* was related to tempeh *β*-glucan content, which also increased (Figure 3). These results are consistent with the research conducted by Rizal et al. [14] that increasing the number of these two microorganisms escalates the *β*-glucan content, thus increasing the antibacterial activity of tempeh. As stated by Hetland et al. [13], *β*-glucans are compounds that are antagonistic to several microorganisms, including bacteria, mold, yeast, and viruses.

The increasing antibacterial activity of tempeh during fermentation is also caused by the increase in the number of soy isoflavones. Kustyawati et al. [6] showed that soybeans added with *S*. *cerevisiae* and *R*. *oligosporus* contained daidzein and genistein of approximately 225 and 465, respectively. Increasing the amount of isoflavones increases the inhibitory activity against bacteria because isoflavones act as antimicrobials [30].

Antimicrobial activity is a very important study for food safety and human health. Therefore, it will be very interesting to study the antimicrobial activity of tempeh against other foodborne pathogens.

## 4. Conclusions

The addition of *S*. *cerevisiae and R*. *oligosporus* as mixed inoculums in tempeh fermentation resulted in higher growth of yeast and fungi, *β*-glucan formation, and antibacterial activity of tempeh than without the addition of yeast. Therefore, the tempeh can have better functional properties as healthy food due to the health benefits of *β*-glucan. *In vivo* studies need to be done to prove the effect of adding both microorganisms on improving tempeh's functional properties in mice.

## Data Availability

All data generated during and/or analyzed during the current study are not publicly available because the data are still needed to complete a doctorate program of the first author but are available from the corresponding authors on reasonable request.
